# Targeted Maternal Chagas Disease Screening Among Individuals Born in a Chagas-Endemic Country

**DOI:** 10.1001/jamanetworkopen.2024.49120

**Published:** 2024-12-02

**Authors:** Alvaro Proaño, Neil C. Shah, Sneider A. Gutierrez Guarnizo, Monica Miranda-Schaeubinger, Michael Z. Levy, Robert H. Gilman, Dustin D. Flannery

**Affiliations:** 1 Division of Neonatology, Children’s Hospital of Philadelphia, Philadelphia, Pennsylvania; 2 Department of Pediatrics, University of Pennsylvania Perelman School of Medicine, Philadelphia; 3 Rowan-Virtua School of Osteopathic Medicine, Stratford, New Jersey; 4 Department of International Health, Johns Hopkins Bloomberg School of Public Health, Baltimore, Maryland; 5 Department of Radiology, Children’s Hospital of Philadelphia, Philadelphia, Pennsylvania; 6 Department of Biostatistics, Epidemiology and Informatics, University of Pennsylvania, Philadelphia

## Abstract

This cohort study examines the prevalence of Chagas disease among Hispanic pregnant women delivering in Philadelphia, Pennsylvania.

## Introduction

Chagas disease is a neglected tropical disease caused by the parasite *Trypanosoma cruzi* that affects 6 million people worldwide.^1,2^ In the United States, Chagas is estimated to affect 43 000 women of reproductive age.^1^ Pregnant persons with Chagas are at risk for congenital transmission to the newborn, which accounts for nearly a quarter of all new cases.^3^

In 2022, a US multidisciplinary expert working group^4^ developed recommendations for increased clinician-directed screening for Chagas. They concluded that women of reproductive age who were born in endemic countries should be tested due to vertical transmission risk. In 2024, Clark and Bern^2^ outlined similar recommendations and endorsed prenatal visits as an opportunity to screen pregnant people.

There is a paucity of maternal prevalence data to inform an optimal screening strategy.^4^ Thus, we sought to evaluate the maternal seroprevalence of Chagas disease among persons who presented for childbirth in Philadelphia who identified as Hispanic and were born in an endemic country.

## Methods

This retrospective cohort study was conducted with biobank serum samples collected from patients at hospital admission for childbirth from April 2020 to August 2023 at 2 academic birth hospitals in Philadelphia, Pennsylvania, serving a diverse population. We included a convenience sample of available serum from patients who self-identified as Hispanic and reported birth in a Chagas-endemic country.^5^ Race, ethnicity, and country of origin were self-reported and obtained from the electronic health record. Only the first maternal sample was included for repeat childbirth encounters. The institutional review board at the University of Pennsylvania approved this study as minimal risk and granted a waiver of informed consent. This report follows the STROBE guideline. Given that there is no criterion standard for Chagas testing, we screened based on a recently proposed algorithm^4^; we tested for *Trypanosoma cruzi*–specific immunoglobulin G antibodies using 2 US Food and Drug Administration–approved seroassays. First, all samples were tested using the Hemagen Chagas kit. The Wiener Chagatest recombinant version 3.0 was performed on all positive samples as a confirmatory test. If the first test was positive and the second test was negative, we performed the rapid immunochromatographic assay, Chembio Chagas Stat-Pak.^6^ Deidentified demographic and clinical characteristics were abstracted from the medical record.

## Results

During the study period, 28 698 unique patients presented for childbirth, of whom 2966 (10.3%) identified as Hispanic. Of these, 828 (27.9%) were born in a Chagas-endemic country. The final denominator was 645 patients. The median (IQR) age was 31 (25-35) years, and the largest proportions of patients were born in Mexico (162 [25.1%]), Honduras (164 [25.4%]), and Guatemala (104 [16.1%]). Demographic and clinical characteristics are shown in Table 1, and all maternal countries of birth are shown in Table 2.

**Table 1.** Maternal Characteristics

Maternal characteristic	Sample, No. (%) (N = 645)
Age, median (IQR), y	31 (25-35)
Race	
American Indian	2 (0.3)
Asian	0
Black	53 (8.2)
Mixed	17 (2.6)
Pacific Islander	2 (0.3)
White	366 (56.7)
Unknown or patient declined	68 (10.5)
Other^a^	137 (21.2)
Insurance status	
Public	509 (78.9)
Private	119 (18.4)
Other	17 (2.6)

^a^ Race was categorized per the study data dictionary; thus, further details were unavailable.

**Table 2.** Chagas-Endemic Countries of Origin

Country^a^	Patients, No. (%) (N = 645)	Estimated US Chagas prevalences based on country of origin, %	Estimated prevalence among pregnant people, %
Argentina	5 (0.8)	3.64	9
Belize	3 (0.5)	0.33	NA
Bolivia	1 (0.2)	18.3	29
Brazil	24 (3.7)	0.61	1
Chile	7 (1.1)	0.70	3
Colombia	29 (4.5)	0.51	3
Costa Rica	1 (0.2)	0.17	NA
Ecuador	71 (11.0)	1.38	NA
El Salvador	27 (4.2)	1.90	4
Guatemala	104 (16.1)	1.13	4
French Guiana, Guyana, Suriname	0	0.84	NA
Honduras	164 (25.4)	0.65	1
Mexico	162 (25.1)	0.73	3
Nicaragua	6 (0.9)	0.52	NA
Panama	5 (0.8)	0.52	NA
Paraguay	0	2.13	9
Peru	19 (2.9)	0.44	1
Uruguay^b^	1 (0.2)	0.24	NA
Venezuela	16 (2.5)	0.71	1

Abbreviation: NA, not applicable.

^a^ For sources used to determine which countries were included, the US Chagas prevalence based on country of origin, and the prevalence of Chagas among pregnant people, see the eAppendix in Supplement 1.

^b^ Since 1997, Uruguay has been considered free of the vector; however, congenital transmission can still occur (eAppendix in Supplement 1).

None of the samples were positive for Chagas (0%; 95% CI, 0%-0.6%). Seven samples were positive for the Hemagen test (1.1%; 95% CI, 0.4%-2.2%).

## Discussion

In this retrospective evaluation of maternal Chagas disease in a pregnant Philadelphia cohort based on self-reported ethnicity and country of birth, we found no confirmed cases of Chagas disease. These results suggest that the prevalence of Chagas disease is very low in our maternal population or that combined self-reported ethnicity and country of birth is an insufficient screening method.

Chagas is a major neglected disease, although the prevalence in at-risk populations in the United States remains unclear. Identifying infected pregnant persons is crucial, given that congenital Chagas disease has a high cure rate if detected early.^3,4^ More evidence is needed to develop a reliable and cost-effective screening strategy during pregnancy, given the differences in testing performance.^3^

The study’s strengths include the large sample size over more than 3 years. The study also has limitations. We used a convenience sample from 2 centers in one urban location. We relied on self-reported ethnicity and birth country, and not all countries are represented equally, with half of our samples obtained from pregnant people who were born in countries with lower prevalence.^1^

## Notes

Supplement 1. **eAppendix.** Sources for Countries of Origin and Prevalence

Supplement 2.
Data Sharing Statement
