# Supplementary material for: Targeted Maternal Chagas Disease Screening Among Individuals Born in a Chagas-Endemic Country
Source: JAMA Netw Open. 2024 Dec 2;7(12):e2449120. doi: 10.1001/jamanetworkopen.2024.49120 (PMC11612833; doi:10.1001/jamanetworkopen.2024.49120)
Supplement: Supplement 1. — eAppendix. Sources for Countries of Origin and Prevalence [file jamanetwopen-e2449120-s001.pdf]

Supplemental Online Content

Proaño A, Shah NC, Gutierrez Guarnizo SA, et al. Targeted maternal Chagas disease screening among individuals born in a Chagas-endemic country. *JAMA Netw Open.* 2024;7(12):e2449120. doi:10.1001/jamanetworkopen.2024.49120

eAppendix. Sources for Countries of Origin and Prevalence

This supplemental material has been provided by the authors to give readers additional information about their work.

© 2024 Proaño A et al. *JAMA Network Open.*

eAppendix. Sources for Countries of Origin and Prevalence.

The list of countries identified as Chagas-Endemic was adapted from the World Health Organization.[1] The estimated US Chagas prevalence based on country of origin was adapted from Irish et al.[2] Note that these prevalences are overall prevalence estimates. The estimated prevalences of Chagas disease in pregnant women is based on Santana et al.[3]

[1]World Health Organization, Chagas disease in Latin America: an epidemiological update based on 2010 estimates. *Wkly Epidemiol Rec*. Feb 6 2015;90(6):33-43.
[2]Irish A, Whitman JD, Clark EH, Marcus R, Bern C. Updated estimates and mapping for prevalence of Chagas disease among adults, United States. *Emerg Infect Dis*. Jul 2022;28(7):1313-1320.
[3]Santana KH, Oliveira LGR, Barros de Castro D, Pereira M. Epidemiology of Chagas disease in pregnant women and congenital transmission of *Trypanosoma cruzi* in the Americas: systematic review and meta-analysis. *Trop Med Int Health*. 2020 Jul;25(7):752-763.

© 2024 Proaño A et al. *JAMA Network Open.*
