# Supplementary material for: Targeted Maternal Chagas Disease Screening Among Individuals Born in a Chagas-Endemic Country
Source: JAMA Netw Open. 2024 Dec 2;7(12):e2449120. doi: 10.1001/jamanetworkopen.2024.49120 (PMC11612833; doi:10.1001/jamanetworkopen.2024.49120)
Supplement: Supplement 2. — Data Sharing Statement [file jamanetwopen-e2449120-s002.pdf]

Data Sharing Statement

Proaño. Targeted Maternal Chagas Disease Screening Among Individuals Born in a Chagas-Endemic Country. *JAMA Netw Open*. Published December 02, 2024.
doi:10.1001/jamanetworkopen.2024.49120

Data
Data available: No

Additional Information
Explanation for why data not available: Deidentified data are available upon reasonable request.
